# Oral *Trypanosoma cruzi* Transmission Resulting in Advanced Chagasic Cardiomyopathy in an 11-Month-Old Male

**DOI:** 10.1155/2020/8828950

**Published:** 2020-10-16

**Authors:** Melissa S. Nolan, Joseane Elza Tonussi Mendes, Andres Ricardo Perez Riera, Gabriel Zorello Laporta

**Affiliations:** ^1^Arnold School of Public Health, University of South Carolina, Columbia, SC, USA; ^2^Setor de Pós-graduação, Pesquisa e Inovação, Centro Universitário Saúde ABC, Fundação ABC, Santo André, São Paulo, Brazil; ^3^Clínica do Coração, Cruzeiro do Sul, Acre, Brazil

## Abstract

Our case report describes the youngest clinical acute Chagas disease case and their unusual disease presentation of cardiac failure. In parts of the Brazilian Amazon, cultural practices include weaning infants from breastmilk to solid foods with açaí consumption serving as an intermediary. This practice could place infants at an increased risk of oral *Trypanosoma cruzi* infection and severe Chagasic cardiac disease.

## 1. Case Presentation

On January 6, 2018, a 5-month-old male presented with a 4-day history of fever to the emergency room at the Juruá Regional Hospital and was diagnosed with *Plasmodium falciparum* by microscopy. The patient was treated following the Brazilian Ministry of Health national treatment guidelines [1]: 3 days of twice daily Coartem (fixed combination of artemether (20 mg) and lumefantrine (120 mg)).

**Figure 1:**
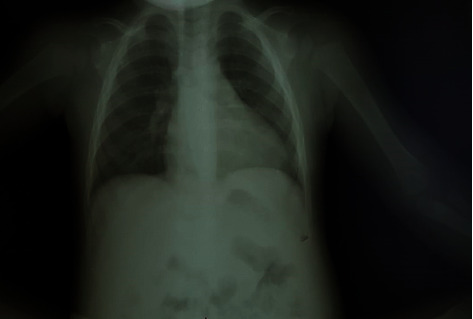
Posteroanterior chest radiograph depicting cardiothoracic ratio >50% by cardiac silhouette.

On June 25, 2018, the now 11-month-old male presented again to Juruá Regional Hospital with fever, upper abdominal pain, and shortness of breath. Anemia (hemoglobin = 10.9 g/dL) (anemia classification was unknown as mean corpuscular volume was not measured) and liver impairment (AST = 44 U/L; ALP = 134 U/L) were noted on blood exams. Thick blood smear microscopy showed *Trypanosoma cruzi* (*T. cruzi*) positive, and the patient was hospitalized. Furthermore, Chagas serology confirmed infection with an immunofluorescent test result of 1/160 IgG titration. After admission, a chest X-ray, electrocardiogram, and upper abdomen ultrasound were ordered. Chest X-ray confirmed cardiomegaly from an enlarged cardiac silhouette (cardiothoracic ratio = 62%) noted on a posteroanterior chest radiograph (Figure 1).

**Figure 2:**
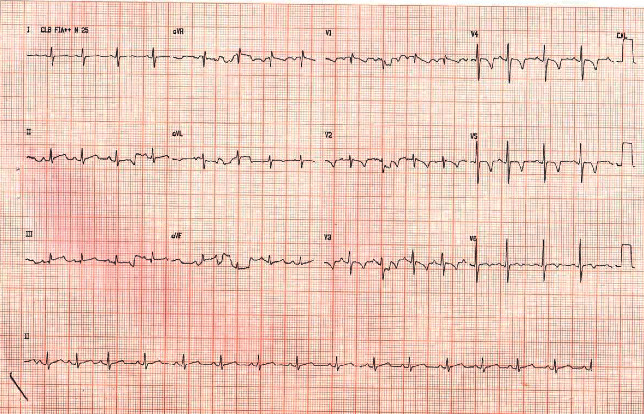
Electrocardiogram depicting electrocardiographic abnormalities.

Electrocardiogram identified regular sinus rhythm (heart rate = 100 bpm), p-wave abnormalities suggestive of right atrial overload, and evidence of right ventricular overload (Figure 2). Hepatosplenomegaly was identified by ultrasound. The patient was diagnosed with “acute form of Chagas disease with cardiac involvement,” and a 60-day dosage of benznidazole in suspension was initiated. During the patient's hospitalization, he took a twice daily 0.8 mL suspension of 25 mg tablets, tolerated the medication well, and exhibited normal liver enzymes on blood examination. After nine days, the patient made marked clinical improvement and was discharged home with a benznidazole prescription.

On July 20, 2018, a 2D doppler echocardiogram with color flow was completed by the sole cardiologist in the region. Echocardiogram demonstrated significant right and left chamber enlargement, mild pericardial effusion, and a mild impairment (LVEF < 50%). The patient was given a once intravenous dosage of dobutamine and started on a daily cardiac failure treatment regimen: captopril, carvedilol, and furosemide. A follow-up appointment with his pediatrician on August 21 indicated the resolution of symptoms and lack of pathological findings on physical examination.

## 2. Discussion

This case report describes the youngest *T. cruzi* clinical case from suspected oral transmission and an unusual presentation of cardiac failure in an infant. This case originated from a rural Amazonian village in the region of Cruzeiro do Sul, Acre state, Brazil, a region with documented oral transmission outbreaks [2]. Local cultural practices include açaí juice consumption which began around 6 months of age to transition infants away from exclusive breastfeeding. Given the lack of palpebral edema or chagoma noted in the patient's history and presenting illness, known as Chagas disease acute cases in the region, and consumption of a commonly contaminated source, oral acute *T. cruzi* transmission was suspected. Congenital transmission cannot be ruled out, as the mother was not tested at the time of the child's diagnosis; however, the attending physician noted that the mother appeared healthy and was no longer breastfeeding. Fortunately, prompt antiparasitic treatment was administered likely preventing mortality, although sadly cardiac failure was not prevented in this 11-month-old male.

Chagas disease typically presents as a chronic organomegaly later in life, after decades of myocardial or smooth muscle tissue alteration and fibrotic damage [3]. Vectoral transmission in childhood traditionally presents as nonspecific flu-like illness with or without chagoma or palpebral swelling. In contrast, orally acquired *T. cruzi* infections demonstrate higher symptomatic rates, graver infections, and a higher number of cardiac involvement than vectoral acute infections [4–6]. Orally acquired *T. cruzi* infection from contaminated food products was first described in the 1960s [7], but a large outbreak stemming from a Venezuelan school [4] raised attention to the public health importance of this transmission source. Contaminated bacaba palm fruit juice, açaí pulp, and other food/beverage products have been linked to oral transmission outbreaks [6, 8–10]. In Venezuela and Brazil, oral *T. cruzi* outbreaks have been increasing over the past decade [5, 6, 11], thought to be linked to increased açaí commercial production in the region [12].

The pathophysiology of Chagas disease clinical progression is complex, largely multifactorial, and likely varies from patient to patient [3]. Higher parasite loads consumed in contaminated food/beverage products likely results in the increased pathogenicity of this transmission source [13]. Infecting parasite discrete typing unit and site of host tissue uptake are also theorized to influence the virulence of oral, acute disease [14, 15]. Another possible factor in our case's clinical severity progression was his prior malarial infection. *Plasmodium falciparum* has been demonstrated to alter chemokine expression [16], which could impact subsequent parasitic infections acquired within a narrow timeframe. Additionally, fill and receipt of the remaining benznidazole dosage was not documented. Given the family's financial strain, it is possible that the patient never completed the 60-day antiparasitic dosage, and the parasitic infection was never fully cleared allowing for continued pathology.

A large ECG study during a recent oral transmission outbreak suggests children are more likely than adults to experience acute electrocardiographic changes as a result of acute oral infection [5]. The permanent nature of the cardiac damage from our patient's acute myocarditis is unusual; however, animal studies support the development of myocardial collagen and fibrotic tissue from oral infection routes [16], providing biological plausibility to our case's clinical manifestation. Furthermore, a follow-up study of Venezuelan acute Chagas disease cases found that while benznidazole eliminated parasitemia, antiparasitic treatment had little effect on progressive myocardial processes eight months later [17]. A large multicenter prospective study of children with all cause myocarditis found that 39% received heart failure therapy one year postresolution of their initial myocarditis [18]. Therefore, one would expect to see more children needing heart failure medications after acute *T. cruzi* infections. The occurrence of these cases in low-resource settings likely influences the lack of prospective cardiac follow-up. Regardless, our case and the literature support the need to follow pediatric acute Chagas disease cases despite benznidazole receipt.

While ECG abnormalities are somewhat frequent in pediatric populations, cardiac failure has rarely been described in literature from vectoral and/or congenital transmission sources. A 7-month-old child with suspected vectoral transmission presented with acute Chagas myocarditis rapidly proceeding to death: this case never received Benznidazole, and diagnosis was not made until postmortem [19]. A 7-year-old child with vectoral transmission developed Chagas cardiomyopathy 3 months after a known triatomine exposure (chagoma presence); however, the exact timing of initial *T. cruzi* infection was unknown and speculated to have previously occurred due to epidemiologic family data [20]. A surveillance study in Mexico identified 14 cases aged 5–18 years with a history of vectoral transmission that presented with cardiomyopathy [21], providing further evidence that children can develop cardiomegaly within a short duration after infection. Lastly, a Bolivian congenital transmission study identified 11% of *T. cruzi*-positive newborns had cardiomegaly noted by chest radiography [22]. These collective cases suggest that children can develop cardiomegaly within a short timeframe, and it is imperative that all pediatric cases be followed for advanced clinical disease progression [23].

Overall, our case report demonstrates that infants can develop cardiomyopathy as a result of orally contaminated *T. cruzi* infection. Our case report makes evident the value of cardiac follow-up of acutely infected children after treatment. Finally, açaí products have been associated with multiple outbreaks in the Amazon region, and public health interventions requiring pasteurization should be implemented to prevent additional Chagas disease cases.

## Data Availability

Our clinical case report is subject to privacy protection, and patient data are not available beyond which is listed in the current manuscript.
